# A Promising Amphotericin B Derivative Induces Morphological Alterations, Mitochondrial Damage, and Oxidative Stress In Vitro and Prevents Mice from Death Produced by a Virulent Strain of *Trypanosoma cruzi*

**DOI:** 10.3390/microorganisms12061064

**Published:** 2024-05-24

**Authors:** Ignacio Martínez, Lucio Rivera-Santiago, Karla Daniela Rodríguez-Hernández, Arturo Galván-Hernández, Lourdes Rodríguez-Fragoso, Lucero Díaz-Peralta, Lisset Torres-Martínez, Lourdes Teresa Agredano-Moreno, Luis Felipe Jiménez-García, Iván Ortega-Blake, Bertha Espinoza

**Affiliations:** 1 Laboratorio de Estudios Sobre Tripanosomiasis y Leishmaniasis, Departamento de Inmunología, Instituto de Investigaciones Biomédicas, Universidad Nacional Autónoma de México, México City 04510, Mexico; imm@iibiomedicas.unam.mx (I.M.); lucio.rivera82@gmail.com (L.R.-S.); xandy411@comunidad.unam.mx (K.D.R.-H.); lisset_torres@ciencias.unam.mx (L.T.-M.); 2 Instituto de Ciencias Físicas, Universidad Nacional Autónoma de México, Apartado Postal 48-3, Cuernavaca 62251, Morelos, Mexico; arturo@icf.unam.mx (A.G.-H.); lucero@icf.unam.mx (L.D.-P.); ivan@icf.unam.mx (I.O.-B.); 3 Facultad de Farmacia, Universidad Autónoma del Estado de Morelos, Av. Universidad, 1001 Col. Chamilpa, Cuernavaca 62210, Morelos, Mexico; mrodriguezf@uaem.mx; 4 Facultad de Ciencias, Universidad Nacional Autónoma de México, México City 04510, Mexico; agredano-moreno@ciencias.unam.mx (L.T.A.-M.); luisfelipe_jimenez@ciencias.unam.mx (L.F.J.-G.)

**Keywords:** A21 amphotericin B derivative, oxidative damage, protection against murine death

## Abstract

Chagas Disease is a neglected tropical disease caused by the protozoan parasite *Trypanosoma cruzi,* affecting 6–8 million people, mainly in Latin America. The medical treatment is based on two compounds, benznidazole and nifurtimox, with limited effectiveness and that produce severe side effects; consequently, there is an urgent need to develop new, safe, and effective drugs. Amphotericin B is the most potent antimycotic known to date. A21 is a derivative of this compound with the property of binding to ergosterol present in cell membranes of some organisms. In the search for a new therapeutic drug against *T. cruzi*, the objective of this work was to study the in vitro and in vivo effects of A21 derivative on *T. cruzi*. Our results show that the A21 increased the reactive oxygen species and reduced the mitochondrial membrane potential, affecting the morphology, metabolism, and cell membrane permeability of *T. cruzi* in vitro. Even more important was finding that in an in vivo murine model of infection, A21 in combination with benznidazole was able to reduce blood parasitemia, diminish the immune inflammatory infiltrate in skeletal muscle and rescue all the mice from death due to a virulent *T. cruzi* strain.

## 1. Introduction

The protozoan parasite *Trypanosoma cruzi* is the etiologic agent of American trypanosomiasis or Chagas Disease (CD), transmitted principally to humans by insect vectors (family Hemiptera; subfamily Triatominae). This infection affects around 6-8 million people worldwide, being endemic in Latin America and representing a serious and economic global health threat. Vaccines against *T. cruzi* are lacking, and the current chemotherapy, based on benznidazole (Bz) and nifurtimox (Nf), shows limited effectiveness in the chronic stage of the disease and produces severe collateral effects, which often result in treatment interruption. Therefore, developing novel and effective drugs against the parasite is crucial, and it includes different strategies [1,2].

Drug repositioning involves finding novel therapeutic molecules from “old drugs”, including approved, withdrawn, abandoned, and investigational drugs. This method has been an exciting strategy to search for new therapeutic solutions for neglected and rare conditions. Today, it is a broadly applied approach to develop innovative and safe drug derivates, enhancing absorption, distribution, metabolism, excretion, and toxicity (ADMET) properties as well as novel action mechanism information, thus significantly shortening development timeframes and cost [3].

The antimycotic compound Amphotericin B (AmpB) was approved for clinical use in 1959 by the U.S. Food and Drug Administration (FDA) to treat invasive fungal infections such as that induced by *Candida* spp. The molecule has shown anti-fungal and anti-protozoan activity [4]. This compound is a polyene whose structure comprises a mycosamine group and a macrolactone ring with seven linked double bonds that connect to the main ring via a glycosidic bond. It has an amphiphilic character, hydrophobic (polyene hydrocarbon chain), and hydrophilic (polyhydroxy chain) properties, being practically insoluble in water.

The mechanisms of action of AmpB are not entirely elucidated. For instance, there is evidence of fungal cell damage by binding and sequestration of the ergosterol, inducing membrane pore formation and oxidative damage [5]. However, AmpB is not currently the first choice of treatment against *T. cruzi* because it has been established that high doses are required, which are toxic to mammalian cells. Therefore, various works have evaluated new formulations of this molecule to develop more effective and less toxic therapeutic options [6,7].

In a previous report, one AmpB amide derivative (A21) was reported, including chemical synthesis, electrophysiology, pharmacology, toxicology, and molecular dynamics. The molecular structure is shown in Supplementary Figure S1. This compound shows an increased selectivity over ergosterol-containing membranes compared to cholesterol-containing membranes. The preclinical and toxicological tests showed that this derivative is just as potent as AmpB but has increased safety. A21 has the same mode of action as AmpB (pore formation in the cell membrane) on *Candida albicans*, believed to be due to structural differences with ergosterol-containing membranes [8]. Moreover, in vivo studies have shown that A21 demonstrates an excellent margin of safety and can be used in antifungal dermal clinical studies; toxicity was observed only in high and repeated doses for long periods [9].

Since ergosterol is one of the main components of the cell membrane of *T. cruzi* [10], the present study aims to characterize the trypanocidal effect induced by A21 and elucidate the mechanism of action in vitro on epimastigotes of a Mexican virulent *T. cruzi* strain (Queretaro, DTU-I), as well as to evaluate its effects in vivo in a murine model of trypanosomiasis.

## 2. Materials and Methods

### 2.1. Chemicals

For in vitro assays commercial Bz, AmpB and Thiazolyl blue tetrazolium bromide (MTT) were purchased from Sigma (St. Louis, MO, USA). Alternatively, for in vivo assays, a suspension of Bz was prepared from commercially available tablets, containing 100 mg of Bz (batch OL0001; Roche, Buenos Aires, Argentina), as previously described [11]. Briefly, tablets of 100 mg Bz were crushed in a mortar and dissolved directly in 10 mL of DMSO (Sigma, USA) [12]. The mixture was gently shaken for 15 min and then centrifuged at 1000× *g* for 10 min. The clear phase containing the Bz was recovered and stored at room temperature until use. The purity of the Bz obtained from the tablet and it’s in vitro trypanocidal activity were compared against the commercial Bz using mass spectrometry.

The AmpB amide derivative A21 (Batch MLO-1053) was synthetized as reported previously [13].

### 2.2. Parasites and Mammalian Cells

Epimastigotes of *T. cruzi* Mexican strains Queretaro (TBAR/MX/0000/Queretaro; DTU-I, Qro), Ninoa (MHOM/MX/1994/Ninoa), Ver6 (MDID/MX/1991/Ver6), and international reference strains Silvio and CL Brener, were cultured in a liver infusion tryptose medium (LIT), supplemented with 10% FBS and 25 µg/mL of hemin and maintained at 28 °C for 3–4 days to obtain the growth log phase, as reported previously [14]. Promastigotes of the *Leishmania mexicana* Bricaire strain, generously donated by Dr. Paulino Tamay (Autonomous University of Campeche, México), were cultured in a M199 medium supplemented with 10% FBS and 2.5 µg/mL of hemin and maintained at 28 °C for 2–3 days to obtain the promastigote growth log phase used for experimental procedures. Procyclic trypomastigotes of the *T. brucei* strain 29-13, generously donated by Dr. Santiago Martinez-Calvillo (FES-Iztacala, UNAM, México), were cultured at 28 °C in an SDM-79 medium, supplemented with 10% fetal bovine serum, as reported previously [15].

Vero cells (monkey kidney epithelium) were maintained in a culture in a complete DMEM medium (10% SFB, 100 IU penicillin, 0.1 mg/mL streptomycin, 0.1 mM non-essential amino acids, 2 mM glutamine, 1 mM sodium pyruvate) at 37 °C and 5% CO_2_. For maintenance, 75–90% confluent cultures were washed with 5 mM Ethylenediaminetetraacetic acid (EDTA), incubated for 5 min with trypsin (1 mg/mL), diluted, and re-plated. Cell-derived Qro trypomastigotes were obtained from Vero cells, infected as described previously [14]. Cell-derived trypomastigotes were collected by centrifugation at 3000× *g* for 10 min and immediately used in the trypanoicidal or infection assays.

### 2.3. Effect of A21 on the Number and Mobility of T. cruzi

To establish the effect of A21 on *T. cruzi* Qro epimastigotes and cell-derived trypomastigotes, 4 × 10^5^ parasites/well were seeded in 96-well plates in the presence of A21 ranging from 0.312 to 10 μM for 6 h. The total number and mobile and non-mobile parasites were established by counting in a Neubauer chamber, using a Microstar IV microscope (Reichert, Depew, NY, USA). As controls, Bz (10 μM), AmpB (0.3 μM) or DMSO (0.26%) were included. Three independent assays in duplicate were performed.

### 2.4. Metabolic Activity of Trypanosomatids

The metabolic activity of various trypanosomatids incubated with A21 was established by the MTT assay. For this, 6 × 10^6^ parasites of several *T. cruzi* strains, *L. mexicana* and *T. brucei*, were incubated with A21 (0.6–10 μM) for 6 h in 60 μL of LIT medium. Twelve μL of MTT (5 mg/mL) were added to reach a final concentration of 0.83 mg/mL and incubated for 5 h at 28 °C. Subsequently, the excess of MTT was removed by centrifugation at 1000× *g* for 10 min and 100 µL of DMSO was added to dissolve the formazan crystals. Absorbance was established at 595 nm with a 655 nm reference filter in an iMARK microplate reader (Bio-Rad, Hercules, CA, USA). The metabolic activity (%) was determined by the formula (absorbance of treated cells/absorbance of untreated cells) × 100. The mean inhibitory concentration (IC_50_) was calculated using the formula described previously [16]. Three independent assays were performed in duplicates.

### 2.5. Effect of A21 on Intracellular Amastigotes

Five hundred Vero cells in 20 µL of DMEM/well were seeded in a 21-well slide (Electron Microscopy Science, Hatfield, PA, USA) and left to reach 1000 cells per well, adhering for 24 h. The medium was then removed and 10,000 cell-derived trypomastigotes were added in 20 µL of complete DMEM, to have a Multiplicity of Infection (MOI) 10:1 (parasite:cell). They were incubated for 6 h, washed twice with PBS and 20 µL of medium alone or containing A21 (6.25–100 μM), AmpB (5 μM) or Bz (10 μM) were added and incubated for 48 h at 37 °C and 5% CO_2_. The medium was removed from each well and after washing twice with PBS, the cells were fixed with 20 μL of methanol for 5 min and stained with Giemsa (Bayer, Leverkusen, Germany). Briefly, 20 μL of Giemsa (0.075%, methanol 6.5%, glycerol 3.5%) were added to each well for 10 min, washed twice with 20 μL of PBS pH 7.1, and finally washed in distilled water by 30 s. The slides were mounted with Organo/limonene mount (Santa Cruz Biotechnology, Dallas, TX, USA) and examined with a microscope model DME (Leica, Wetzlar, Germany) at 40×. The number of amastigotes per 100 cells in each condition was established. Three assays were performed in duplicate.

### 2.6. Cytotoxic Effect of A21 in Vero Cells and Selectivity Index

To establish the cytotoxic effect of A21 on mammalian cells, 2 × 10^3^ Vero cells/ well in 100 µL were seeded in a 96-well microplate and kept at 37 °C and 5% CO_2_ for 24 h. A21 (3–100 µM), AmpB (5 μM) or benznidazole (10 μM) were added and the cells were incubated for 48 h at 37 °C and 5% CO_2_. Metabolic activity was determined adding MTT to reach a final concentration of 0.45 mg/mL and incubated for 5 h at 37 °C. The excess of MTT was removed by gentle vacuum and 115 µL of DMSO was added to dissolve the formazan crystals. The metabolic activity (%) and cytotoxic concentration (CC_50_) were determined as above for IC_50_.

The Selectivity Index (SI) was calculated, comparing the cytotoxicity in mammalian cells (CC_50_) and the trypanocidal activity (IC_50_): CC_50_ Vero/IC_50_ parasite. If the value obtained is greater than 1, the compound will have a preferential effect on parasites and not on mammalian cells [17].

### 2.7. Effect of A21 in Morphology and Ultrastructure of T. cruzi

The induced morphological changes in *T. cruzi* by A21 were established by Giemsa stain and Transmission Electron Microscopy (TEM). Briefly, epimastigotes were incubated with A21 (5 and 10 μM) for 6 h at 28 °C. They were harvested by centrifugation at 1000× *g* for 10 min, and washed two times with PBS, then 1.6 × 10^4^ parasites/5 μL/well were air-dried on a 21-well slide. Immediately, they were fixed with methanol (20 μL), stained with Giemsa and mounted as described above. The parasites were examined with a microscope model DME (Leica, Wetzlar, Germany) at 100× [17].

For TEM, 30 × 10^6^ of A21-treated epimastigotes (5 μM for 6 h) were fixed with paraformaldehyde 4%-glutaraldehyde 2.5% in PBS (pH 7.2). Post-fixation was made in 1% osmium tetroxide for 2 h and embedded in propylene oxide and Epon resin (1:1) for 18 h. Ultrathin sections (40–60 nm) were obtained, contrasted with uranyl acetate 5% and lead citrate 0.5% for 20 min, and observed on TEM in a JEOL1010 (JEOL, Peabody, MA, USA), operating at 80 kV.

### 2.8. Cell Volume Determination

Epimastigotes Qro strain (7.5 × 10^6^/150 µL) were cultured in 96-well cell plates, treated with A21 (5 or 10 μM) or medium alone (negative control), and incubated for 30 or 60 min. Subsequently, the change in cell volume was evaluated by changes in absorbance at 490 nm as reported previously for other trypanosomatids [18], using a microplate reader model 550 (Biorad, Hercules, CA, USA). The results were normalized with respect to the absorbance value of untreated parasites.

### 2.9. Change in Cell Membrane Permeability

To establish whether the parasites treated with A21 undergo changes in membrane permeability that compromise the integrity of the parasite, Qro epimastigotes (2 × 10^6^/mL) were incubated for 30 or 60 min with 5 or 10 μM of A21. Parasites were centrifuged at 1000× *g*, washed as described above, and resuspended in 498.5 µL of buffer (50 mM HEPES, 700 mM NaCl, 12.5 mM CaCl_2_, pH 7.4), and 1.5 µL of propidium iodide (PI) (Invitrogen, Carlsbad, CA, USA) (1.5 mM). Samples were incubated for 20 min at room temperature and immediately analyzed in a FACSCalibur flow cytometer (Beckton Dickinson, Franklin Lakes, NJ, USA) with a 670 nm/long-pass filter (FL3-H) for PI (red fluorescence/dead cells). A total of 20,000 events per treatment were acquired. The data were analyzed using FlowJo 7.3.2 software and expressed as the percentage of cells for each population phenotype (PI+). Heat-killed parasites (55 °C for 10 min) were used as a positive control [14].

### 2.10. Determination of Intracellular Oxidative Stress

The induction of intracellular oxidative stress produced by A21 was assessed by the detection of reactive oxygen species (ROS) using the oxidant-sensitive fluorescent probe DCFDA (Sigma, USA). Epimastigotes (2 × 10^6^/mL) were incubated with A21 (5 or 10 μM) for 30 and 60 min. Treated parasites were harvested, washed, and resuspended in cold PBS for 45 min in the dark with 10 μM DCFDA at 28 °C. Epimastigotes treated with 160 µM H_2_O_2_ were used as a positive control, as reported previously [19]. The oxidation of DCFDA was determined by the fluorescent product 2, 7 dichlorofluorescein (DCF in a Sinergy H1 microplate reader (Biotek, Winooski, VT, USA) (λex of 488 nm and λem of 530 nm). Results were expressed as the fluorescence DCF+ arbitrary units (AU) mean of values obtained for treated and untreated (control) cells, respectively [14].

### 2.11. Mitochondrial Potential Assay (ΔΨm)

Epimastigotes (2 × 10^6^/mL) incubated with A21 (5 or 10 μM) for 30 or 60 min were washed with PBS as above, resuspended in 0.5 mL of PBS with 10 μg/mL Rhodamine 123 (Rho 123) (Sigma, USA) and incubated for 20 min at room temperature. Parasites treated with CCCP 100 µM (Sigma, USA) were used as a positive control of mitochondrial depolarization. Then, alterations in the fluorescence intensities for Rho 123 (FL1-H) were quantified by the Index of Variation (IV) that was obtained using the equation (TM − CM)/CM, where TM is the median of fluorescence for treated parasites and CM is the median of fluorescence for control parasites (untreated). Negative values of IV correspond to the depolarization of the mitochondrial membrane [19].

### 2.12. Ergosterol Quantification

To quantify ergosterol, a modification of the spectrophotometric procedure previously described for fungi was carried out [20]. Briefly, 1.2 × 10^8^ epimastigotes of *T. cruzi* Qro strain in 3 mL of culture were incubated in the presence of A21 (5 or 10 µM) or Bz (10 µM), at 28 °C for 6 h. Then, epimastigotes were centrifuged at 3000× *g* for 10 min. A wash was performed with PBS and the centrifugation was repeated. The wet weight was established and 1 mL of 25% potassium hydroxide (0.25 g of KOH + 0.35 mL of sterile distilled water, brought to 1 mL with 100% ethanol) was added for every 200 mg of wet weight. It was mixed in a vortex for 20 s and incubated in a thermocycler (MJ Research, Waltham, MA, USA) for 30 min at 85 °C. Then, it was allowed to cool at room temperature and 0.4 mL of water and 1 mL of n-heptane were added per each 200 mg of initial wet weight. The tubes were vortexed for 20 s. Finally, they were allowed to stand at room temperature for 1 h and the n-heptane (upper layer) was recovered. The absorbance was recorded at 230 and 281 nm, using an NP1000 nanodrop (UV-VIS function). Ergosterol content was calculated as previously described [20].

### 2.13. Effect of A21 on In Vivo Infection Model

To establish if A21 had an effect in vivo on *T. cruzi*, groups of six eight-week-old female Balb/c mice were infected with 1 × 10^5^ blood trypomastigotes of *T. cruzi* Qro strain, as previously reported [21]. From the first day post-infection (dpi), A21 (40 mg/kg) was administered intraperitoneally alone or in combination with Bz (40 mg/kg) for 24 days. As controls, infected untreated mice were included, as well as mice uninfected and treated with the same compound doses. During the course of the experiments, weight, piloerection and movement were evaluated as indications of the general health of the mice [22]. The weight of the animals was measured every week. Spontaneous voluntary locomotor activity was evaluated by direct observation every day for 1 min in the morning and afternoon and the exploratory activity was classified as good (+++), reduced (++), or scarce (+). The presence of piloerection was also established through direct observation of each animal during the treatment.

Parasitemia was evaluated every third day for 90 dpi; for this, 5 µL of blood was taken from the caudal vein and diluted 1:20 in PBS to be examined in a Neubauer chamber under an optical microscope (MJ Research, USA). Mortality was determined daily. Additionally, the presence of pale areas (chalky white patches) on the skeletal muscle of the posterior extremities, characteristic of infection with *T. cruzi* Qro strain, previously described in our model, was checked in the treated and infected mice [21].

### 2.14. Histological Analysis

Representative heart and skeletal muscle samples were collected in the acute (18–21 dpi) and chronic (90 dpi) phases of infection. Heart and skeletal muscle samples were fixed in 4% formalin solution for 48 h and processed as reported previously [21]. Samples (2 µm) were stained with Hematoxylin-Eosin (H-E) for histological examination using an optical microscope.

### 2.15. Mice and Animal Ethical Management

Eight-week-old female Balb/c mice were purchased from the Biological Models Unit at IIB, UNAM, and maintained at their facilities. Food and water were provided ad libitum. All experiments that involved animals followed the ethical guidelines of the Instituto de Investigaciones Biomédicas, Universidad Nacional Autónoma de México (https://www.biomedicas.unam.mx/wp-content/pdf/intranet/reglamentos/codigo-etico-iibo.pdf?x30807) (accessed on 8 March 2024). Institutional Commission for the Care and Use of Laboratory Animals; approval code: ID 150; approval date: 30 July 2021.

### 2.16. Statistical Analysis

All experiments were performed in duplicates or triplicates, in at least two independent experiments. Significant statistical differences were established by one-way ANOVA with Tukey‘s multiple comparisons with a 95% confidence level. All statistical tests were performed using GraphPad Prism 5.0 (GraphPad Software, San Diego, CA, USA). The data were expressed as mean plus standard deviation (SD), and differences were considered statistically significant when *p* < 0.05.

## 3. Results

### 3.1. Compound A21 Reduces the Number and Mobility of T. cruzi

**Figure 1:**
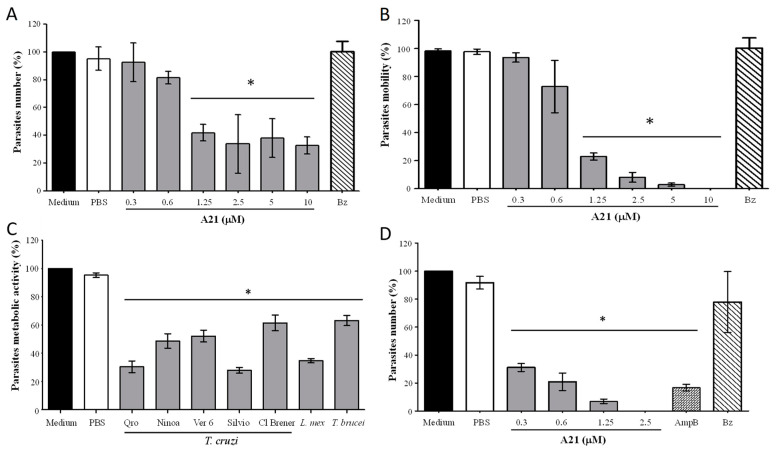
Effect of A21 on different trypanosomatids. (**A**) Number of parasites and (**B**) motility were established in *T. cruzi* Qro epimastigotes incubated for 6 h with several concentrations of A21 or benznidazole (Bz, 10 μM) and observed in Neubauer chamber by optical microscopy. (**C**) *T. cruzi* epimastigotes of several strains, *T. brucei*, or *L. mexicana* promastigotes were incubated for 6 h with A21 (2.5 μM) and metabolic activity was measured by the MTT assay, as described in Materials and Methods. (**D**) *T. cruzi* cell-derived trypomastigotes were incubated for 6 h with several concentrations of A21, Amphotericin B (AmpB, 0.3 μM) or Bz (10 μM) and the number of parasites was determined by observation in a Neubauer chamber by optical microscopy and expressed as percentage. In all cases, medium alone or PBS were included as controls. Three independent experiments were performed with duplicates. Values represent means ± the standard deviation. Significant differences by one-way ANOVA with Tukey’s post test (* *p* < 0.05) are indicated with respect to parasites in medium.

When evaluating the effect of A21 on the Qro *T. cruzi* in vitro, it was observed that the total number and mobility of the epimastigotes was significantly reduced at 6 h incubation at concentrations from 1.25 μM to 10 μM (Figure 1A,B). This does not occur when using the reference drug Bz (10 μM). Compound A21 also reduces the metabolic activity of the Qro epimastigotes when incubated for 6 h. This effect was also observed in other strains of *T. cruzi* and in other trypanosomatids species such as *L. mexicana* and *T. brucei* with 2.5 μM of A21 (Figure 1C). It was found that, with the exception of Cl Brener, all *T. cruzi* strains, *L. mexicana* and *T. brucei* had an IC_50_ between 1.26 and 3.2 μM (Table 1).

A21 also (6 h) reduces the number of cell-derived trypomastigotes in vitro, like the effect of AmpB (Figure 1D). When comparing the effect of A21 on epimastigotes and trypomastigotes, it was observed that the latter were more sensitive to the compound on the number of parasites (Figure 1D). Likewise, the selectivity index is higher when evaluating the effect of A21 on trypomastigotes, compared to epimastigotes (Table 1).

### 3.2. A21 Reduces the Number of Intracellular Amastigotes

**Figure 2:**
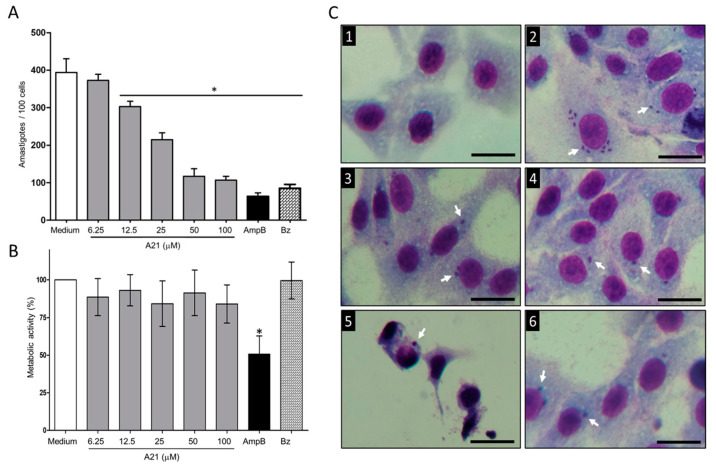
Effect of A21 on *T. cruzi* amastigotes and Vero cells. (**A**) The infection assay in Vero cells was performed as described in Materials and Methods, in the absence or presence of different concentrations of A21, AmpB (5 μM) or Bz (10 μM) for 48 h. Cells were fixed and stained with Giemsa. The number of amastigotes per 100 cells was counted by optical microscope at 40×. (**B**) Effect of A21, AmpB and Bz on the metabolic activity of Vero cells. The test was performed as described in Materials and Methods. Values represent means ± the standard deviation. Significant differences by one-way ANOVA with Tukey’s post test (* *p* < 0.05) are indicated with respect to parasites in medium. (**C**) Photographs of uninfected and untreated cells (**1**), infected and untreated cells (**2**), treated with 50 μM A21 (**3**) and 100 μM (**4**), 5 μM AmpB (**5**) or 10 μM Bz (**6**). Amastigotes are indicated by white arrows. Scale bar = 50 μm.

When evaluating the effect of A21 on the intracellular Qro amastigotes, a reduction in the number was observed in a dose-dependent manner (Figure 2A). The reduction in the number was achieved from 12.5 μM to 100 μM of A21, with an IC_50_ of 27.56 μM. These concentrations were not cytotoxic to Vero cells up to 48 h of incubation (Figure 2B). The reduction in the number of intracellular parasites was observed without morphological changes in the Vero cells treated with A21 confirming the compound’s low cell toxicity. In contrast, severe morphological changes were observed when the infected cells were treated with AmpB. This may be the cause of the lower number of amastigotes, since many cells were damaged, and their internal parasite lost (Figure 2C). Even with the highest concentration tested (100 µM) the Vero metabolic activity CC_50_ was not reached.

With the data obtained, the SI was calculated, and it was found that for Qro trypomastigotes it was >300. In amastigotes SI was 3.62. For other strains of trypanosomatids, epimastigotes and *L. mexicana* promastigotes SI was greater than 30 (except for CL Brener), which implies that the compound has a better effect on parasites than on mammalian cells (Table 1).

### 3.3. A21 Alters the Morphology and Ultrastructure of T. cruzi

**Figure 3:**
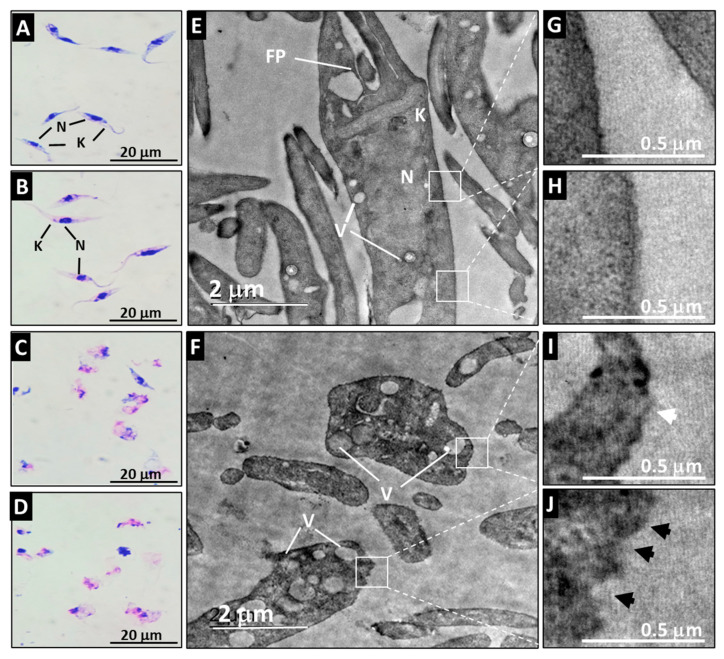
Effect of A21 on the morphology and ultrastructure of *T. cruzi*. Epimastigotes of Qro strain were incubated for 6 h with medium alone (**A**,**E**), PBS (**B**), or in the presence of 5 μM (**C**,**F**) or 10 μM (**D**) of A21. Morphology was established by Giemsa staining (**A**–**D**) in a light microscopy (100×) or TEM (**E**,**F**) as described in Materials and Methods. N (nucleus), K (kinetoplast), FP (flagellar pocket), V (vacuoles). White boxes indicate zoom of the intact membrane of control parasites (**G**,**H**) and the damaged membrane of A21-treated parasites (**I**,**J**), showing disruptions in the continuity of the membrane (white arrowhead) or irregularities in the membrane (black arrowheads). Scale bars are indicated in all cases.

The Qro epimastigotes retained their characteristic morphology when they were incubated with medium or with PBS (Figure 3A,B). Their ultrastructure was also preserved when observed by TEM where the typical shape of the nucleus, kinetoplast and membrane can be appreciated (Figure 3E,G,H). Loss of flagellum, swelling of the cell, and vacuolization of the cytoplasm were evident after A21 treatment (Figure 3C,D). The ultrastructure analyzed by TEM of the A21 treated parasites showed that there is a disorganization of the genetic material, with membrane swelling, membrane disruption and abundant vacuoles and kinetoplast disorganization (Figure 3F,I,J).

### 3.4. A21 Induces Early Changes in Metabolic Activity, Cell Volume and Membrane Permeability in T. cruzi

**Figure 4:**
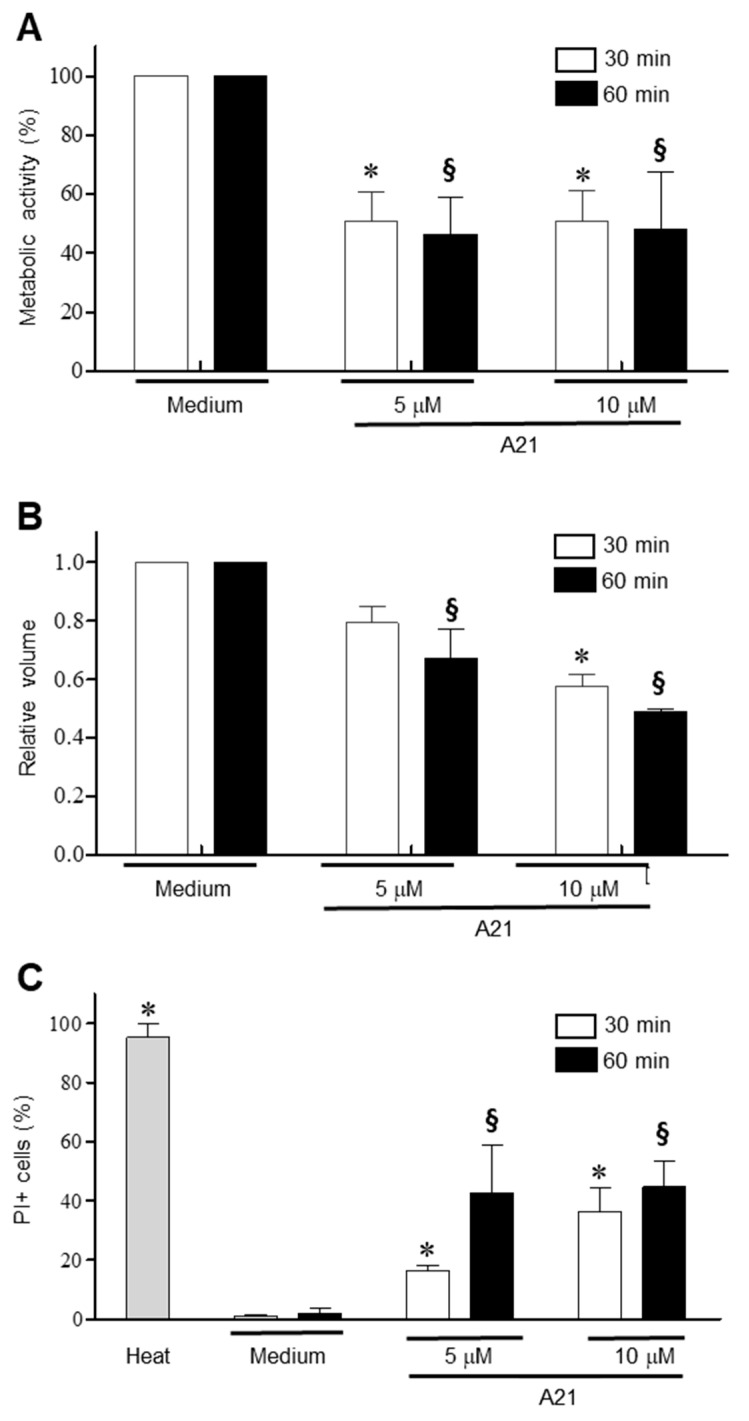
Mechanism of action of A21 on *T. cruzi*. (**A**) Epimastigotes of Qro strain were incubated with A21 (5 or 10 μM) for 30 or 60 min and metabolic activity by the MTT assay was determinate. (**B**) Changes in cell volume in the parasites treated with A21 was established by spectrophotometry. (**C**) The increase in cell permeability by the incorporation of propidium iodide was determined in the epimastigotes treated with A21. Medium alone or heat (65 °C, 10 min) were used as controls. PI: propidium iodide. Values represent means ± the standard deviation. Significant differences by one-way ANOVA with Tukey’s post test (*p* < 0.05) are indicated with respect to parasites in medium at 30 min (*) or 60 min (§).

To establish the early events that A21 triggers on epimastigotes, the parasites were incubated for 30 and 60 min in the presence of 5 and 10 μM of A21 and the change in metabolic activity was established using the MTT assay. There was a significant reduction in metabolic activity with both concentrations and time tested (Figure 4A). Under these same conditions, a decrease in cell volume was observed, dependent on the compound concentration and incubation time (Figure 4B). Membrane damage was studied on *T. cruzi* epimastigotes by PI internalization. PI exhibits a fluorescence enhancement upon binding DNA due to a compromise in membrane integrity. The parasites treated with A21 (5 or 10 μM) showed a concentration and time-dependent increase in the PI+ fluorescence, indicating increased membrane permeability (Figure 4C).

### 3.5. A21 Increases the Reactive Oxygen Species

**Figure 5:**
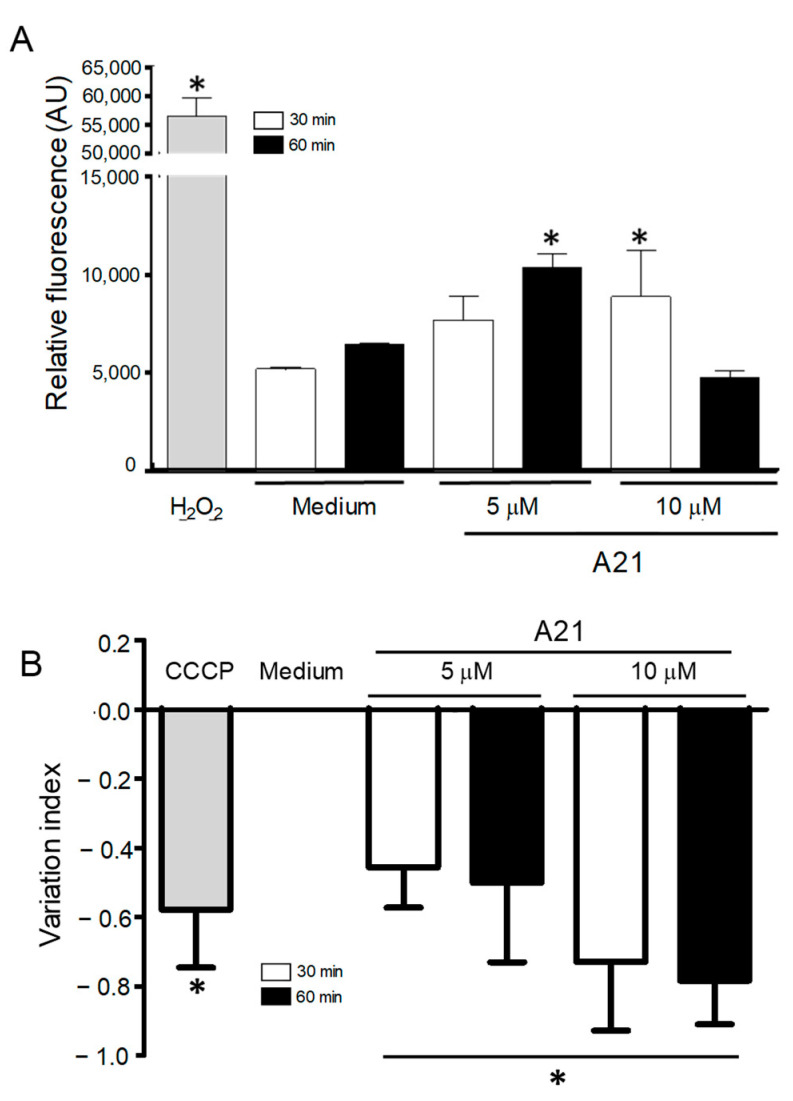
ROS generation and changes in mitochondrial membrane potential, induced by A21. (**A**) Epimastigotes of Qro strain were incubated with A21 (5 or 10 μM) for 30 or 60 min and the generation of ROS was evaluated by the increase in fluorescence of the DCFDA compound. Changes are expressed as arbitrary units (AU); Medium and H_2_O_2_ were used as controls. (**B**) The change in mitochondrial membrane potential was established as described in Materials and Methods and expressed as a variation index. Medium and CCCP (100 μM) were used as controls. Values represent means ± the standard deviation. Significant differences by one-way ANOVA with Tukey’s post test (* *p* < 0.05) are indicated with respect to parasites in medium.

Since one of the mechanisms of action of AmpB is the accumulation of reactive oxygen species (ROS) on fungal cells, we decided to investigate possible oxidative damage caused by A21 in the parasites. The intracellular ROS production was analyzed using the fluorescent probe DCFDA, which fluoresces in the presence of ROS. A21 significantly increased total ROS production in epimastigotes. The parasites treated with 5 μM (60 min) and 10 μM (30 min) showed an increase in ROS production as compared to the negative control (Figure 5A). The concentration of 10 μM of A21 at 60 min showed no difference with the control. The positive control (H_2_O_2_ 160 µM) produced a strong fluorescent signal.

### 3.6. A21 Induce Depolarization of Mitochondrial Membrane Potential (ΔΨm)

Previous studies have reported that AmpB induces ROS in yeast and *Leishmania sp*. mitochondria and inhibits the respiratory chain. To analyzed whether the A21 derivative could induce changes in the mitochondrial membrane potential (∆Ψm) of *T. cruzi* epimastigotes, the cationic dye Rh123 was used. A21 caused a concentration and time-dependent decrease in the mitochondrial membrane potential after 30 and 60 min of treatment compared with the parasite incubated only with the LIT medium (Figure 5B). The loss of ΔΨm was higher in epimastigotes treated with 10 µM of A21 by 30 and 60 min, showing IV values of −0.73 and −0.78, respectively. The control of mitochondrial depolarization, CCCP (100 µM), showed IV values of −0.58.

### 3.7. A21 Affects the Mobility and Reduces Weight in T. cruzi Infected Mice

To establish whether A21 had the capacity to protect mice from the infection of the virulent *T. cruzi* Qro strain in vivo, an infection murine model previously described by our group was used. Balb/c mice were infected with 1 × 10^5^ Qro *T. cruzi* blood trypomastigotes intraperitoneally. Then, from the first dpi, 40 mg/kg of A21 alone, Bz alone or the combination of both were administered intraperitoneally every 12 h for 24 days.

**Figure 6:**
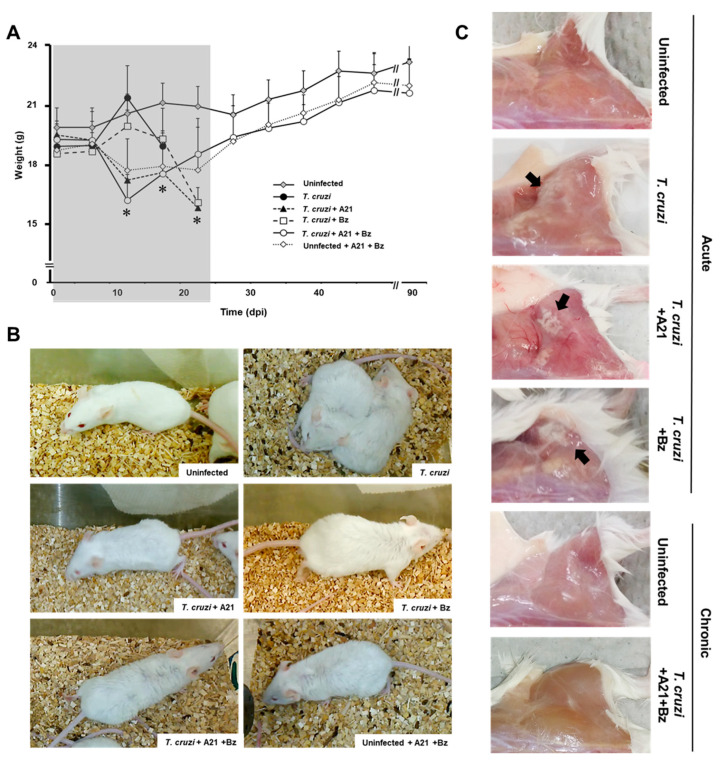
Effect of A21 in an in vivo model. (**A**) Female mice (Balb/c) were inoculated intraperitoneally with 1 × 10^5^ blood trypomastigotes of *T. cruzi* Qro strain. Since day 1 post-infection, A21 (40 mg/kg), Bz (40 mg/kg) or a combination of both, were administered intraperitoneally every 12 h for 24 days and the weight was recorded weekly, until the end of the experiment at 90 dpi. Only the mean and upper SD are presented to avoid visual saturation. Values represent means ± the standard deviation. Significant differences by one-way ANOVA with Tukey’s post test (* *p* < 0.05) are indicated with respect to uninfected mice. Gray shaded area indicates the duration of treatment. (**B**) Piloerection was observed in all groups, except in uninfected, untreated mice (uninfected). (**C**) Mice died during the acute phase or were sacrificed at 90 dpi. They were reexamined for pale areas in skeletal muscle of the posterior legs (black arrows). Representative results of one experiment from a total of two independent experiments are shown.

Weight loss was observed in the mice treated with A21 alone or in combination with Bz from 7 dpi until the end of the treatment (24 dpi). The premature weight loss in A21-treated mice is due to the effect of the compound, since the weight of uninfected drug-treated mice is also reduced by up to 15% (Figure 6A). Once the treatment finished at 24 dpi, the infected mice treated with A21 + Bz and the uninfected mice treated with A21 + Bz gradually recovered their weight. By the end of the experiment, they did not have significant differences with respect to the healthy controls.

Piloerection was also observed in the treated mice, from the second dpi. This macroscopic alteration was maintained until the end of the treatment (24 dpi) (Figure 6B) and subsequently disappeared as the mice reached the chronic stage of the infection.

The pale areas observed in the skeletal muscle of the posterior legs of the infected mice during the acute stage of the infection, which are characteristic of the Qro strain infection, were not found in the mice treated with A21 + Bz that survived to 90 dpi (Figure 6C).

It has previously been established that in the murine model, the infection with the *T. cruzi* Qro strain gradually reduces the mobility and exploratory activity of mice until it leaves them in a state of prostration, followed by death. When the activity of each group was monitored daily inside their cages, it was observed that the treated groups, despite weight loss and piloerection, retained better activity and movement than those infected without treatment (Supplementary Table S1).

### 3.8. A21 in Combination with Benznidazole Reduce Parasitemia and Prevents Death in Mice

In the in vivo assays, Bz obtained from tablets was used, whose composition and purity were evaluated by mass spectrometry. The results showed that the obtained molecule from the tablets is Bz MS [ESI+] 283 *m*/*z* [Bz+Na]+. Likewise, its in vitro effect on *T. cruzi* was similar to that observed with the commercial Bz (Supplementary Figure S2).

**Figure 7:**
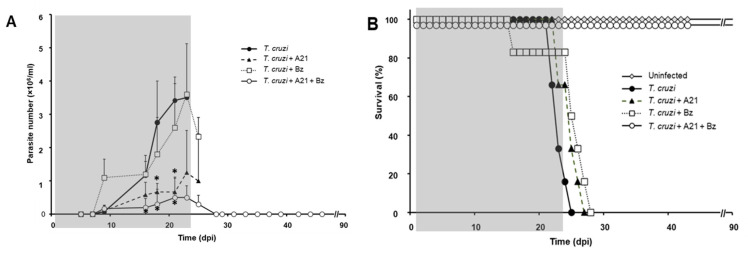
Effect of A21 on parasitemia and survival. (**A**) Female mice (Balb/c) were inoculated with 1 × 10^5^ Qro strain blood trypomastigotes and treated as indicated in Materials and Methods. Parasitemia was evaluated every third day, until the end of the experiment at 90 dpi. Only the mean and upper SD is presented to avoid visual saturation. Statistical analysis by one-way ANOVA with Tukey’s post test (* *p* < 0.05) with respect to the group infected, untreated (*T. cruzi*). (**B**) Survival was recorded daily, until the end of the experiment at 90 dpi. The gray shaded area indicates the duration of treatment. Representative results of two independent experiments are shown.

The blood parasitemia was evaluated every third day and the death of the animals was recorded daily. Uninfected mice, untreated infected mice, and uninfected treated mice were included as controls to monitor the A21 effect on the mice. It was observed that the infection with *T. cruzi* induces the increased presence of parasites in the blood in a time-dependent manner (Figure 7A).

Mice infected without treatment died between 20 and 25 dpi (Figure 7B). Infected mice treated with Bz alone did not change the course of the parasitemia (Figure 7A) and did not prevent the death of the mice (Figure 7B). On the other hand, the administration of A21 reduced parasitemia by up to 85% compared to the untreated infected group at 21 dpi, but did not prevent the death of the mice. Importantly, the combination of A21 with Bz reduced parasitemia and prevented death in all treated mice (Figure 7A,B).

**Figure 8:**
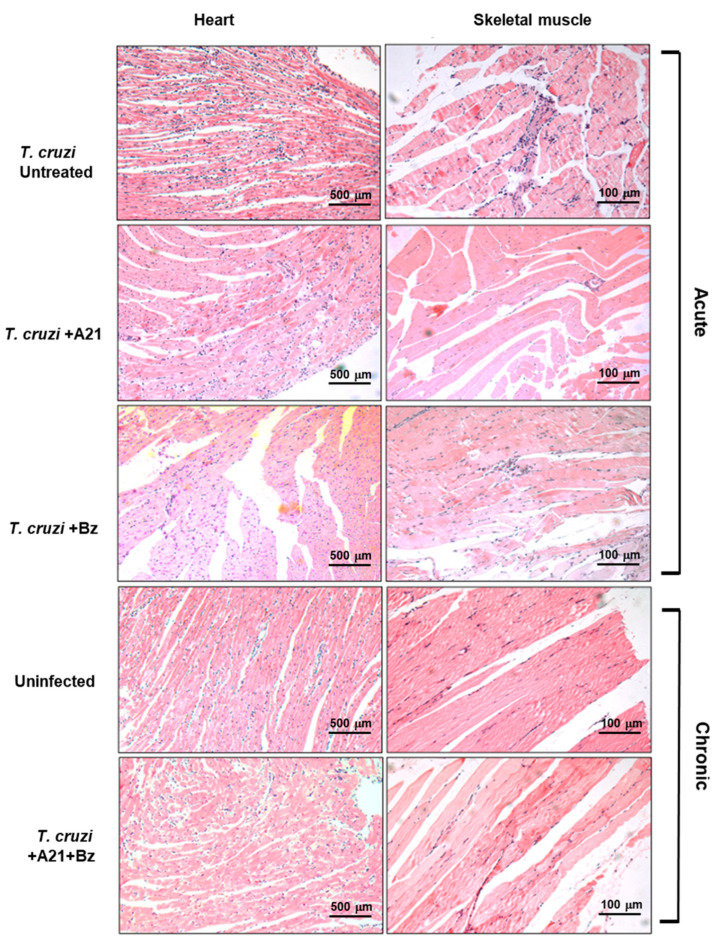
Histopathological analysis of *T. cruzi* infection and treatment in heart and skeletal muscle. Inflammatory infiltrate observed during the acute and chronic phases of infection with *T. cruzi*, in the absence or presence of treatment with A21, benznidazole, or a combination of both. Hematoxylin-Eosin stain. Magnification 10×.

The presence of inflammatory infiltrate was observed in the cardiac tissue of infected mice with *T. cruzi* during the acute phase. Importantly, this infiltrate was lower in the hearts of A21 + Bz-treated mice in the chronic phase. Inflammatory infiltrate observed in the skeletal muscle decreased in the presence of A21 in the acute phase (Figure 8). Also, with the combination of both compounds, a reduction was observed in the skeletal muscle of the mice in chronic phase (Figure 8).

## 4. Discussion

The negative economic and social impact of CD produced by the parasite *T. cruzi* and the problems involved in its treatment make it necessary to search for new drugs to counteract the parasite. Since these organisms have a complex life cycle involving several morphological and functional stages, they encounter diverse environmental stressors to which they must successfully adapt, including responses to new drugs [23].

AmpB has been used for decades to treat fungal infections, it is highly toxic with serious side effects, and its mechanism of action is still not completely understood. Although the primary mechanism of action reported has been binding to ergosterol in cell membranes, resulting in pore formation [24]. The design of new analogues with low toxicity and high selectivity could contribute to the discovery of new treatment options or help with the study of the parasite’s trypanocide mechanism and stress response [8].

A21, a new AmpB derivative, was evaluated on a highly virulent strain of *T. cruzi*. A21 induced a reduction in number and mobility of *T. cruzi* epimastigotes in a few hours in vitro. In addition, A21 reduces the metabolic activity of several *T. cruzi*. strains and other trypanosomatids as *L. mexicana* and *T. brucei*. These data are important since few compounds have been shown to have an effect on more than one trypanosomatid, which implies a broad spectrum of possible use in the future [25,26]. A21 also reduced the number of trypomastigotes in 6 h and they were more sensitive to the effect of the compound than epimastigotes or amastigotes. Likewise, the effect observed at 6 h is an advantage when compared to other compounds that require several days to exert their trypanocidal action [26].

On the other hand, A21 reduces the number of intracellular amastigotes in a manner similar to the effect of AmpB, but without the cytotoxic effect of the latter. It had previously been reported that AmpB reduces the number of intracellular amastigotes of *T. cruzi* in Vero cells, with a dose less than 10 μM [27]. However, the marked cytotoxic effect of AmpB has limited its use as an anti-*T. cruzi* agent. The low cytotoxicity of A21 on mammalian cells makes it an interesting candidate as an anti-trypanosomatids drug.

These results indicate that the sensitivity of the *T. cruzi* Qro strain to compound A21 is higher to trypomastigotes than in epimastigotes and amastigotes. This opens the possibility of using A21 as an alternative to eliminate trypomastigotes in transfusable blood units, which would provide a solution to the current problem regarding the risk of transfusion of contaminated blood, which has not been resolved with other trypanocidal agents [28,29]. This possibility should be explored further.

It has previously been shown that AmpB can form pores in the membrane of *L. mexicana*, facilitating the entry of ions such as K+ and Cl-, which leads to a rounded shape of the parasite, loss of flagellum, depolarization of the mitochondrial membrane and its death [30]. In previous work, A21 has the ability to form pores in the *C. albicans* membrane, allowing the passage of ions such as K+ [8]. When investigating the mechanisms involved in the trypanocidal effect of A21, it was observed that in addition to the changes in cell permeability, there were changes in the morphology, there was an increase in the production of ROS and the consequent depolarization of the mitochondrial membrane. These are events that can induce apoptotic cell death, as has been demonstrated by our group [14].

It remains to be investigated whether the A21 mechanism of action on *T. cruzi* described in vitro in this work is the same for other trypanosomatids such as *Leishmania* sp. and *T. brucei*. However, considering that the latter also have ergosterol in their membrane [31], the possibility of a similar mechanism of action is high.

The trypanocidal effects of various molecules observed in vitro are lost when they are tested in in vivo models or they are as toxic for the parasite as they are for the mammal cells, which represents a filter to finding good candidate molecules for treatment [3].

AmpB, is not the drug of choice against *T. cruzi*. To date, the proposed administration of the compound is limited either in micelles or in liposomal formulation. This has been shown to reduce parasitemia and reduce or delay mortality in some mice models. However, it has not been seen when virulent *T. cruzi* strains are used [7,32]. Unfortunately, the nephrotoxicity and hepatotoxicity of the compound continue to limit its clinical use in patients.

In contrast, A21 proved to be non-toxic in a rat model, which is promising for its clinical use [9]. When the A21 effect in a *T. cruzi* murine model was evaluated, the most significant finding was to demonstrate that the combinate action of A21 and Bz, in doses lower than the commonly used [33], effectively reduced blood parasitemia and mortality induced by a highly virulent strain. It is central to mention that A21 does not present cytotoxicity on mammalian cells in vitro in doses until 400 µM. At the end of the experiments the initial weight loss was recovered, and the mice looked like the healthy, uninfected mice.

Previous work has shown that the use of suboptimal concentrations of Bz (less than 100 mg/kg body weight) in combination with other compounds significantly reduces parasitemia and mortality in mice infected with *T. cruzi.* The effect of the combination is better than the administration of each of the compounds separately [34,35]. Particularly, it has been reported that the combination of Bz with molecules that interfere with ergosterol synthesis reduces parasitemia and favors the survival of infected mice [34]. In our case, a possible explanation for the suppression of *T. cruzi* infection by A21 mixture with Bz is the combined oxidative action on the genetic material and proteins caused by Bz and the addition of the possible binding of A21 to the membrane’s ergosterol causing pores in the *T. cruzi* membrane and allowing the ions to flow out producing parasite death. We also have results that indicate that parasites incubated with A21 have a reduction in the amount of ergosterol detected by a colorimetric assay (Supplementary Table S2). This aspect must be studied further in the future.

Reduced motility and piloerection are events that had been previously reported in infected mice with *T. cruzi* strain Qro, and that compromise muscular function and leg movement [21]. The treatment with A21 in combination with Bz decreased the loss of mobility, which indicates a better state of health of the mouse. Likewise, it prevented the formation of pale areas in the skeletal muscle associated with the infection, which had previously been reported to persist up to 90 dpi when 1 × 10^4^ parasites were inoculated [21]. In this work, 10-fold more parasites were inoculated and, despite this, the pale structures were not found in the muscle at 90 dpi, nor was there histological evidence of muscle regeneration with abundant adipose tissue that was previously reported in this infection model [21]. A preliminary histological study suggests that the combined treatment reduces the inflammatory infiltrate. This aspect must be studied further. 

Possible biases of the study may be the use of only female mice, which could limit the role of hormonal influence, and the use of only one parasite genetic group (DTU I). Also, varied drug concentrations and different treatment periods must be considered in the future. Liposomal carriers are currently being studied to improve A21 disposal.

## 5. Conclusions

The results of the present study indicate that A21 in vitro induces changes in cell membrane permeability, which leads to an intracellular imbalance that increases ROS levels and causes damage to the mitochondrial membrane. These events induce morphological and ultrastructural changes that affect the parasite in a few hours. While in vivo, the combined administration of A21 and Bz reduces parasitemia and damage to muscle tissue, particularly cardiac and skeletal muscle, and diminishes inflammatory infiltrate, preventing the death of the mice, when they are infected with a virulent strain of *T. cruzi*. This made the combination of A21 and Bz a promising option for treatment of this neglected parasitic disease.

## 6. Mexican Patente

Composición farmacéutica conteniendo Beznidazol y N-(L)- histidinamida de Amfoterisina B para el tratamiento de la tripanosomiasis. Mx/a/2019/007278

## Figures and Tables

**Table 1:** Trypanosomatid A21 IC_50_ and SI.

Parasite	Strain	Stage	DTU *	A21 IC_50_ (μM) at 6 h **	Selectivity Index
	Qro	Trypomastigotes	Tc I	<0.3	>333
	Qro	Epimastigotes	TcI	1.26 ± 1.0	>79.36
	Qro	Amastigotes	Tc I	27.56 ± 2.32	>3.62
*T. cruzi*	Ninoa	Epimastigotes	TcI	2.03 ± 1.03	>49.26
	Silvio	Epimastigotes	TcI	0.86 ± 0.1	>116
	Cl Brener	Epimastigotes	TcVI	>10	ND
	Ver 6	Epimastigotes	TcVI	2.86 ± 0.97	>34.96
*L. mexicana*	Bricaire	Promastigotes	----	0.8 ± 0.4	>125
*T. brucei*	29-13	Epimastigotes	----	3.2 ± 0.5	>31.25

* Discreet Typing Unit of *T. cruzi*; ** 48 h for amastigotes. ND: non-determined

## Data Availability

The raw data supporting the conclusions of this article will be made available by the authors on request.

## Supplementary Materials

The following supporting information can be downloaded at: https://www.mdpi.com/article/10.3390/microorganisms12061064/s1, Figure S1: Structure of Amphotericin and A21; Figure S2: Bz analysis and in vitro effect; Table S1: Mobility of mice infected and treated; Table S2: Ergosterol content in *T. cruzi* epimastigotes.
