# Supplementary material for: A Promising Amphotericin B Derivative Induces Morphological Alterations, Mitochondrial Damage, and Oxidative Stress In Vitro and Prevents Mice from Death Produced by a Virulent Strain of Trypanosoma cruzi
Source: Microorganisms. 2024 May 24;12(6):1064. doi: 10.3390/microorganisms12061064 (PMC11205368; doi:10.3390/microorganisms12061064)
Supplement: Supplementary file 1 [file microorganisms-12-01064-s001.zip › Suplementary Tables.docx]

Supplementary Table 1. Mobility of mice infected and treated

| Dpi | 10 | 12 | 14 | 16 | 18 | 20 | 22 | 24 |
| --- | --- | --- | --- | --- | --- | --- | --- | --- |
| No infected | +++ | +++ | +++ | +++ | +++ | +++ | +++ | +++ |
| *T. cruzi* | +++ | +++ | ++ | ++ | ++ | ++ | + | + |
| *T. cruzi* + A21 | +++ | +++ | +++ | ++ | ++ | ++ | ++ | + |
| *T. cruzi* + Bz | +++ | +++ | +++ | ++ | ++ | ++ | ++ | + |
| *T. cruzi* + A21 + Bz | +++ | +++ | +++ | +++ | +++ | ++ | ++ | ++ |

+++ Good exploratory activity, good mobility

++ Reduced exploratory activity, reduced mobility

+ Little exploratory activity, little mobility

Supplementary Table 2. Ergosterol content in *T. cruzi* epimastigotes

|  | Ergosterol content (%)  Mean ± SD |
| --- | --- |
| Medium | 100 |
| A21 0.5 μM | 46.5 ± 6.9* |
| A21 10.0 μM | 24.6 ± 3.0* |
| Bz 10.0 μM | 93 ± 2.8 |

*Significant difference vs. control (medium) p<0.05
