# Supplementary figures and images for: A Promising Amphotericin B Derivative Induces Morphological Alterations, Mitochondrial Damage, and Oxidative Stress In Vitro and Prevents Mice from Death Produced by a Virulent Strain of Trypanosoma cruzi
Source: Microorganisms. 2024 May 24;12(6):1064. doi: 10.3390/microorganisms12061064 (PMC11205368; doi:10.3390/microorganisms12061064)

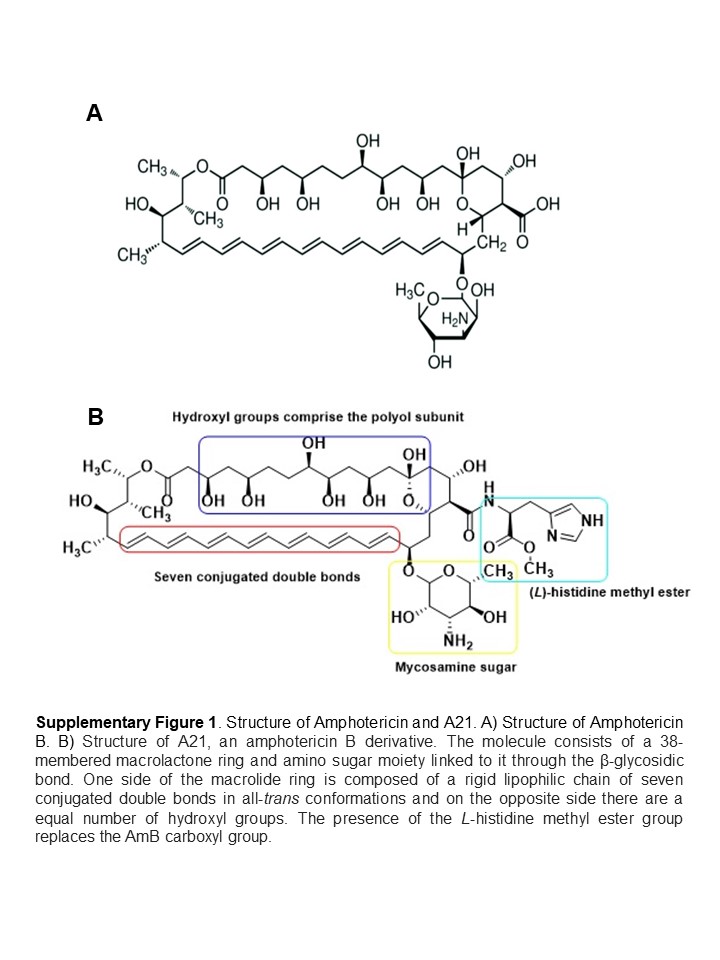

Supplement: Supplementary file 1 [file microorganisms-12-01064-s001.zip › Supplementary Figure 1.jpg]

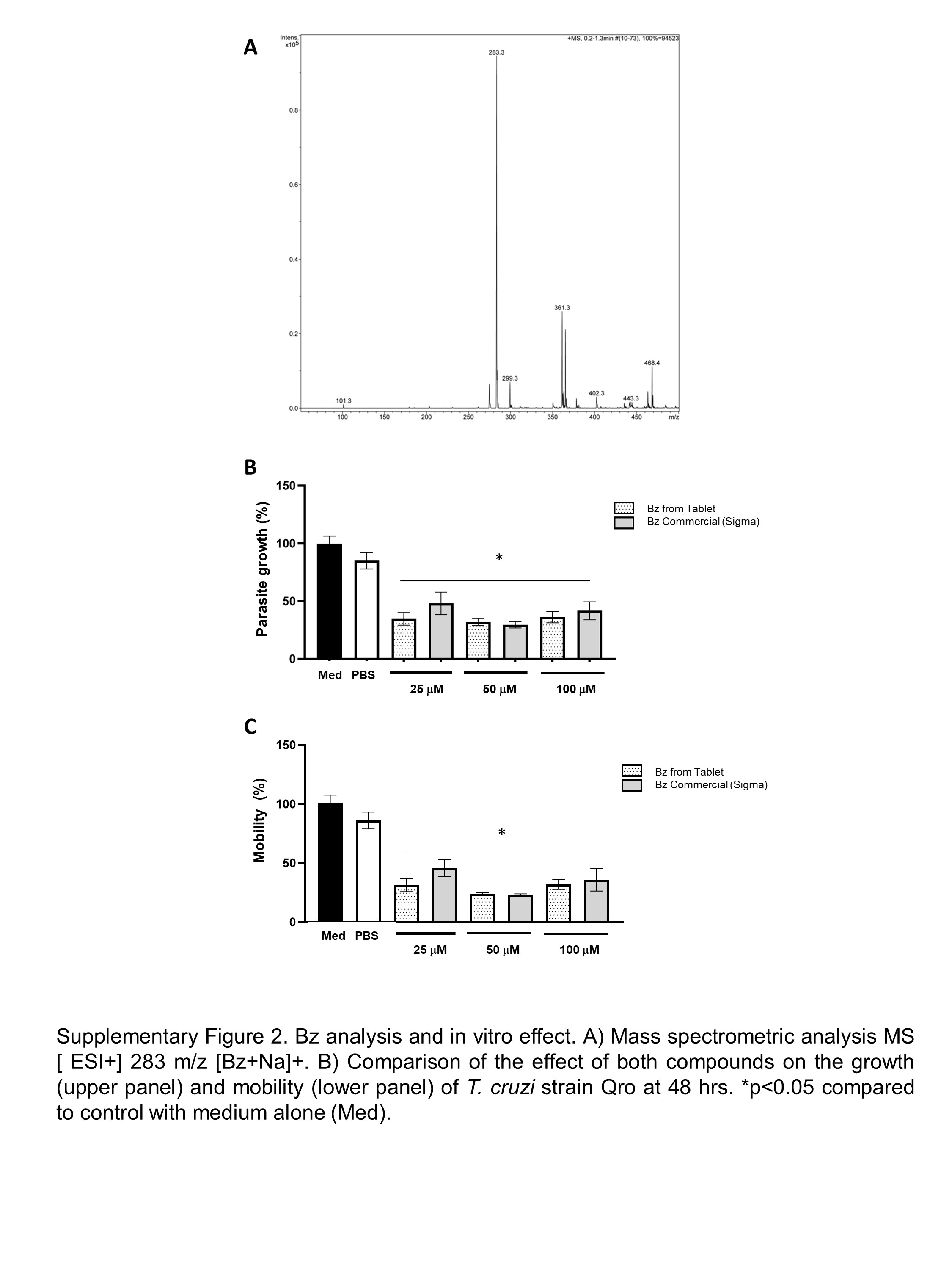

Supplement: Supplementary file 1 [file microorganisms-12-01064-s001.zip › Supplementary Figure 2.jpg]
